# Temperature and Wavelength Dependence of Energy Transfer Process Between Quantized States and Surface States in CdSe Quantum Dots

**DOI:** 10.1186/s11671-017-1971-6

**Published:** 2017-03-24

**Authors:** Lei Zhang, Qinfeng Xu, Mingliang Liu, Lingbin Kong, Mengmeng Jiao, Haifeng Mu, Dehua Wang, Honggang Wang, Jiannong Chen, Chuanlu Yang

**Affiliations:** 1 School of Physics and Optoelectronic Engineering, Ludong University, YanTai, 264025 China; 2 Ministerial Key Laboratory of JGMT, Nanjing University of Science and Technology, Nanjing, 210094 China

**Keywords:** Quantum dots, Quantized states, Surface trap states, Energy transfer (ET)

## Abstract

Temperature and wavelength dependence of energy transfer (ET) process between quantized states and surface trap states of CdSe quantum dots was investigated, respectively. The experimental results demonstrate that the photoluminescence (PL) intensity of the quantized states decreases with respect to the trap state emission, especially at lower temperatures. The observed ET process between quantized states and trap states which is influenced by the thermal population behavior. At the same temperature, the silver films can greatly enhance the energy transfer (ET) rate from the quantized states to trap states due to surface plasmonic coupling effect.

## Background

Colloidal quantum dots (QDs) have displayed extensive applications for QD LEDs [1, 2], QD lasers [3–7], and QD solar cells [8–10]. For some applications, surface trap states of the QDs should be adequately suppressed to obtain high fluorescence efficiency of core quantized states. As a result, many researchers have employed various surface chemistry methods to eliminate surface emission [11]. However, the surface state of QDs also plays an important role in photoluminescence (PL) quantum yield [12], photoluminescence (PL) lifetime [13], multi-exciton recombination [14–16], and optoelectronic processes [17–19]. Recently, some experimental results demonstrate that the emission of surface trap states with the wide visible light range can be applied to white LEDs [20–22]. Hu et al. have realized the fluorescent color tuning by post-synthesis thermal annealing method which enhances the PL intensity of surface trap states for CdSe QDs on polymethyl methacrylate (PMMA) silver films [23, 24].

However, the photoluminescence (PL) properties of surface states remain poorly understood with respect to our understanding of the quantized states. To elucidate the role of surface trap states, the surface state emission has been the focus of many investigations. Lifshitz et al. [25, 26] have confirmed the existence of recombination between shallow trapped electrons and deep trapped holes in the surface trap states of CdSe QDs by magnetic resonance. Wang et al. investigated the up-converted PL process of CdTe QDs at room temperature which confirms the existence of surface states [27]. More importantly, Wang et al. further analyzed the photoluminescence (PL) dynamic process of surface states by time-resolved photoluminescence (PL) measurement at room temperature [28]. We have reported that multi-excitons created in the quantized states can be effectively extracted to the acceptor trap states by utilizing an intrinsic energy transfer system in CdSe nanocrystals (NCs) [29]. One can conclude that simple PL spectroscopy at room temperature does not reveal the nature of the surface states.

D. Kim et al. [30–33] have systematically reported the temperature effect on photoluminescence (PL) dynamics of the CdSe QDs, and the experimental result demonstrates that the bound-exciton state causes a significant impact on radiative decay lifetime. Temperature dependence of thermal population behavior is an important factor for the ET process from quantized states to surface states. However, researches paid little attention to the temperature-dependent dynamics of trap states and ET process between quantized states and trap states. The photoluminescence (PL) mechanism of trap states is being highly debated. Therefore, in order to better understand the photoluminescence (PL) origin of surface trap states in the CdSe QDs, it is important to explore temperature effect on photoluminescence (PL) decay and photoluminescence (PL) spectra of quantized states and surface trap states, respectively. Here, we investigate temperature and wavelength dependence of photoluminescence (PL) properties of surface states and ET process from low temperature to room temperature in this paper.

## Methods

### Synthesize Samples

The CdSe quantum dots were diluted by toluene solution and the diluted CdSe quantum dot solution was added with PMMA powders on the scale of 30:1. In order to dissolve the PMMA powders, the mixed samples were placed in a dark room for several days. Then, the silicon wafer was deposited with 60-nm silver films by thermal evaporation method and the deposition film thickness was checked by the crystal thickness monitor. The PMMA–QD mixture was spin-coated on silicon wafer with 60-nm silver films at a speed of 3000 rpm. Consequently, the PMMA–QD silver film samples were prepared.

### Experiment Apparatus

For PL spectral measurements, the 800-nm picosecond Ti: Sapphire laser with 76 MHz repetition rate was used to generate the 266-nm and 400-nm wavelength pulse laser based on second harmonic and third harmonic conversion technique, respectively. The 266-nm and 400-nm pulse lasers were focused onto the sample surface with a power density of ~100 W/cm^2^ at an incident angle of ~45°. The photoluminescence (PL) from samples was collected vertically by a 60× objective and sent to the spectrometer, and the emission photoluminescence (PL) spectra were recorded with a monochromator (Acton SP-2500i, 0.5 m, 150 lines mm^−1^ grating, blazed at 500 nm). For time-resolved PL decay measurements, the photoluminescence (PL) from the samples was collected by the same objective and then was detected by the single-photon counting system with the 250 ps time resolution. Temperature dependence of PL and lifetime measurements were performed in the liquid helium cryogenic system from low temperature to room temperature.

## Results and Discussion

**Fig. 1:**
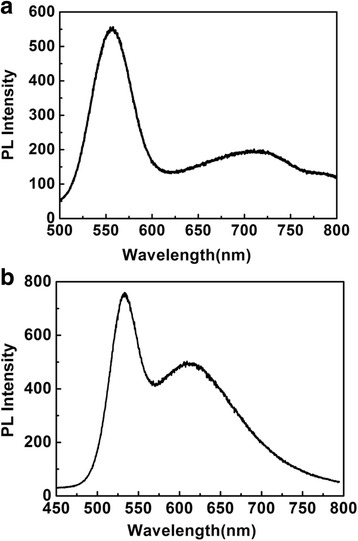
**a** PL spectrum of the CdSe QDs on quartz coverslip. **b** PL spectrum of the CdSe QDs on the PMMA silver films

The PL spectra of quantized states and surface trap states for QDs on quartz coverslip at room temperature are shown in Fig. 1a, which consists of two peaks at 550 and 700 nm for the core quantized states and surface trap states, respectively. From the PL spectra, the PL intensity of quantized states is dominant and the PL intensity of trap states is weak. Figure 1b shows PL spectra of the quantized states and trap states for QDs on the silver films at room temperature which consists of two peaks at the wavelength 525 and 625 nm from the core quantized states and surface trap states, respectively. For QDs on the silver films, the QDs had been coated with PMMA films and then spin-coated on silicon wafer with 60-nm silver films, so that the PMMA films can prevent the energy transfer from quantum dots to silver films which causes the fluorescence quenching effect. As compared with the PL properties of the CdSe on quartz coverslip, the lower PL intensity of quantized states emission and the enhancement PL intensity of trap states emission for QDs on the silver films are observed, which can be attributed to the enhanced carrier recombination in trap states and the enhanced ET rate from the quantized states to the trap states due to plasmonic coupling effect on the corrugated silver films. From the absorption spectra, the PL intensity of trap states became obvious only when the excitation wavelength was tuned within the quantized absorption band and the small surface volume of trap states could prevent their efficient absorption transitions [34, 35]. Therefore, It can be concluded that the energy transfer process should be the origin of the activation energy.

**Fig. 2:**
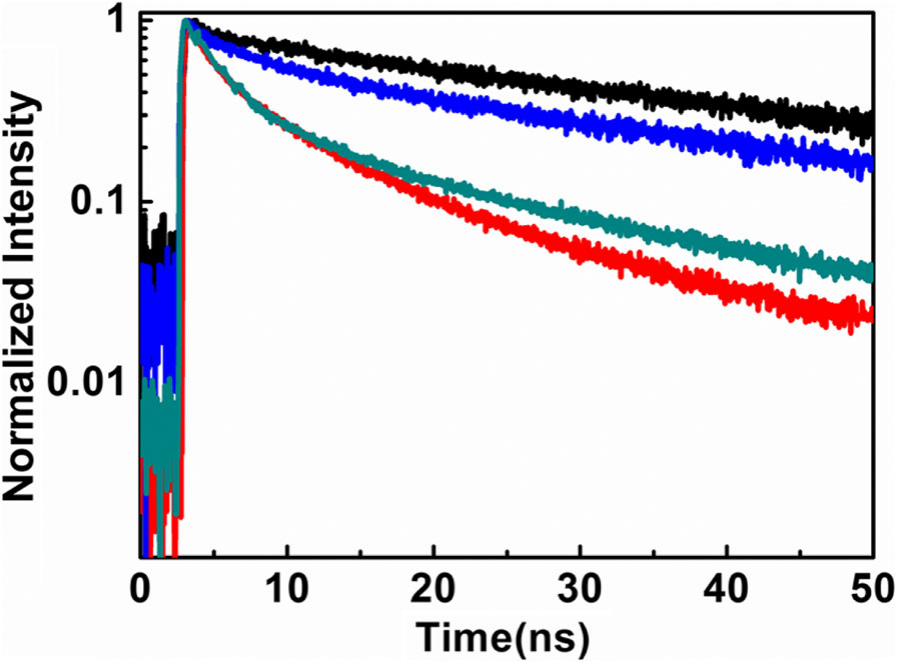
Time-resolved PL decays for the CdSe QDs on quartz coverslip surface and PMMA silver films measured at quantized states emission and trap states emission, respectively. Trap states emission (*black lifetime curve*) and quantized states emission (*green lifetime curve*) on quartz coverslip surface; trap states emission (*blue lifetime curve*) and quantized states emission (*red lifetime curve*) on the PMMA silver films, respectively

To explicate the above steady-state PL measurements, the time-resolved PL measurements of quantized states and trap states were investigated, respectively. The PL decay curves of quantized states and trap states for CdSe QDs on the quartz coverslip and silver films are shown in Fig. 2. The lifetime decay curve of quantized state emission (green line) for CdSe QDs on the quartz coverslip is well fitted with double exponential functions which exhibit a fast component of 2.2 ns and a slow component of 21.1 ns. The fast component can be attributed to the intrinsic energy transfer process between quantized states and surface trap states. And the lifetime decay curve of trap states emission (black line) is fitted with single exponential function which only exhibits a slow component of 61.7 ns as shown in Fig. 2. However, the lifetime decay curve of quantized states emission (red curves) for CdSe on PMMA silver films with a slow component of 16.1 ns and a fast component of 1.6 ns and the lifetime decay curve of trap states emission (blue line) with 26.9 ns are shown in Fig. 2. By the above analysis, the relative faster component corresponds to ET lifetime between quantized states and trap states of semiconductor NCs, which strongly suggests that the surface states capture the excitons through the ET process [36], instead of direct carrier trapping or Auger-assisted carrier transfer through the multi-exciton ionization effect. According to above lifetime comparison, the PL lifetimes of quantized states and trap states on the PMMA silver films become shorter and the corresponding ET rate is greatly enhanced. The results demonstrate that the local surface plasmon from the QD/silver hybrid structures enhances trap state emission and accelerate ET process in QDs.

**Fig. 3:**
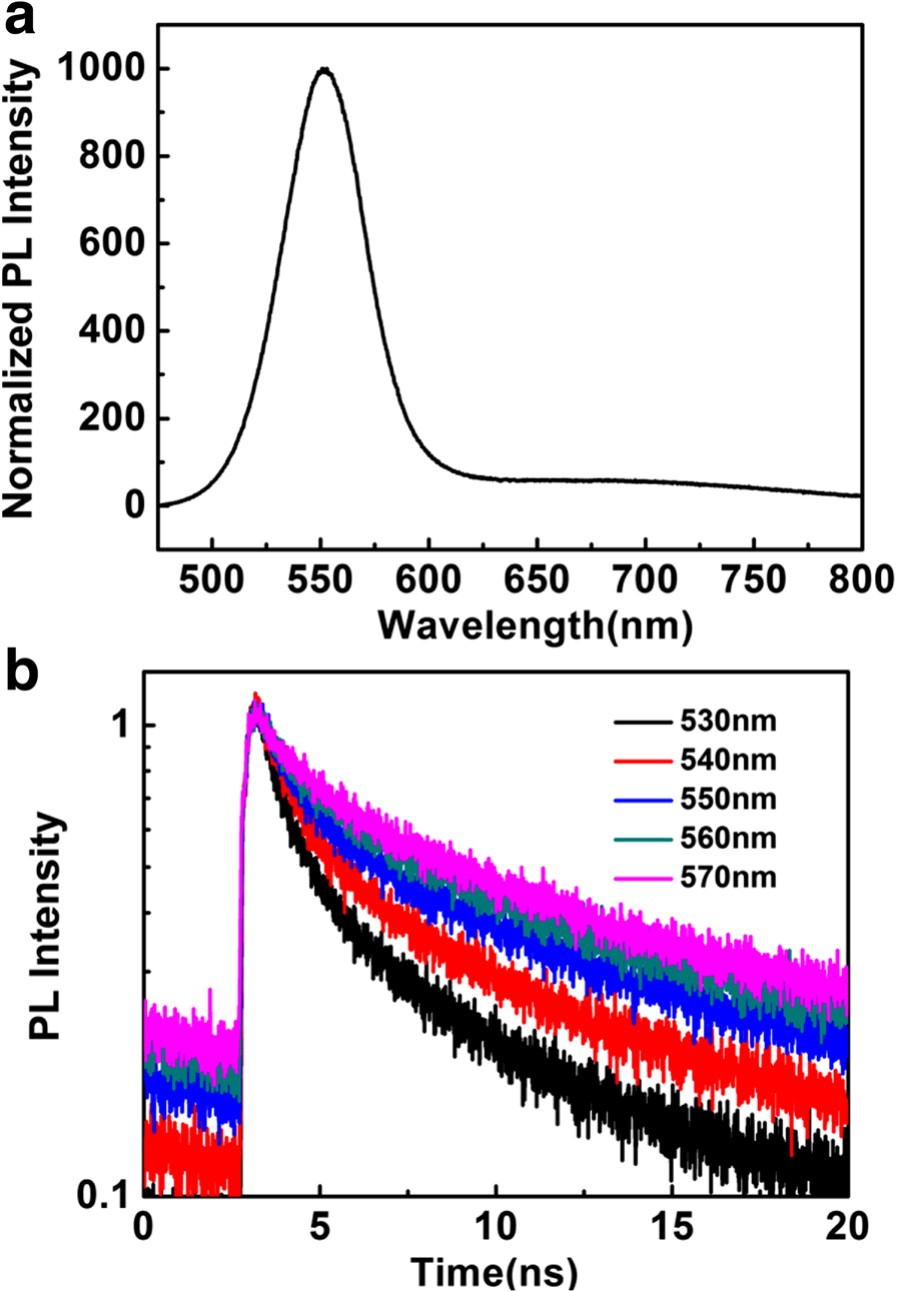
**a** PL spectrum of the CdSe QDs in solution. **b** Different wavelength PL decays of quantized state emission for the CdSe QDs in solution

To further verify ET process from the quantized states to trap states inside QDs and eliminate ET process between QDs, we carried out time-resolved and spectrally resolved PL measurement within narrow spectral bands across the whole emission spectrum of quantized states for QDs in solution. The PL spectrum of CdSe QDs in solution is shown in Fig. 3a. However, the ET process between the QDs is absent in these diluted QD solutions due to the longer distance between the QDs. Figure 3b shows different wavelength PL decay curves of quantized states for CdSe QDs in solution. The PL decay curves were measured at the several emission peaks with 10 nm spectral width for CdSe QDs in solution. The lifetime decay curves are well fitted with double exponential functions which exhibit the fast component of 2.11, 2.25, 2.33, 2.37, and 2.41 ns at the peak value of wavelength 530, 540, 550, 560, and 570 nm, respectively. The results clearly demonstrate that the ET dynamic process is not homogeneous between quantized states and surface trap states, and the exciton decay lifetimes of quantized states become slower from the shorter wavelength to the longer wavelength. It is consistent with the nature of the spectral overlap between the different QDs [37]: for this ET process, the shorter wavelength has a better spectral overlap than the longer wavelength and thus exhibits faster energy transfer process. The spectral dependence of the ET rate is in reasonably good agreement with the steady-state PL measurements. The wavelength-dependent PL decay curves provide clear evidence that the efficient ET process occurs inside the CdSe QDs.

**Fig. 4:**
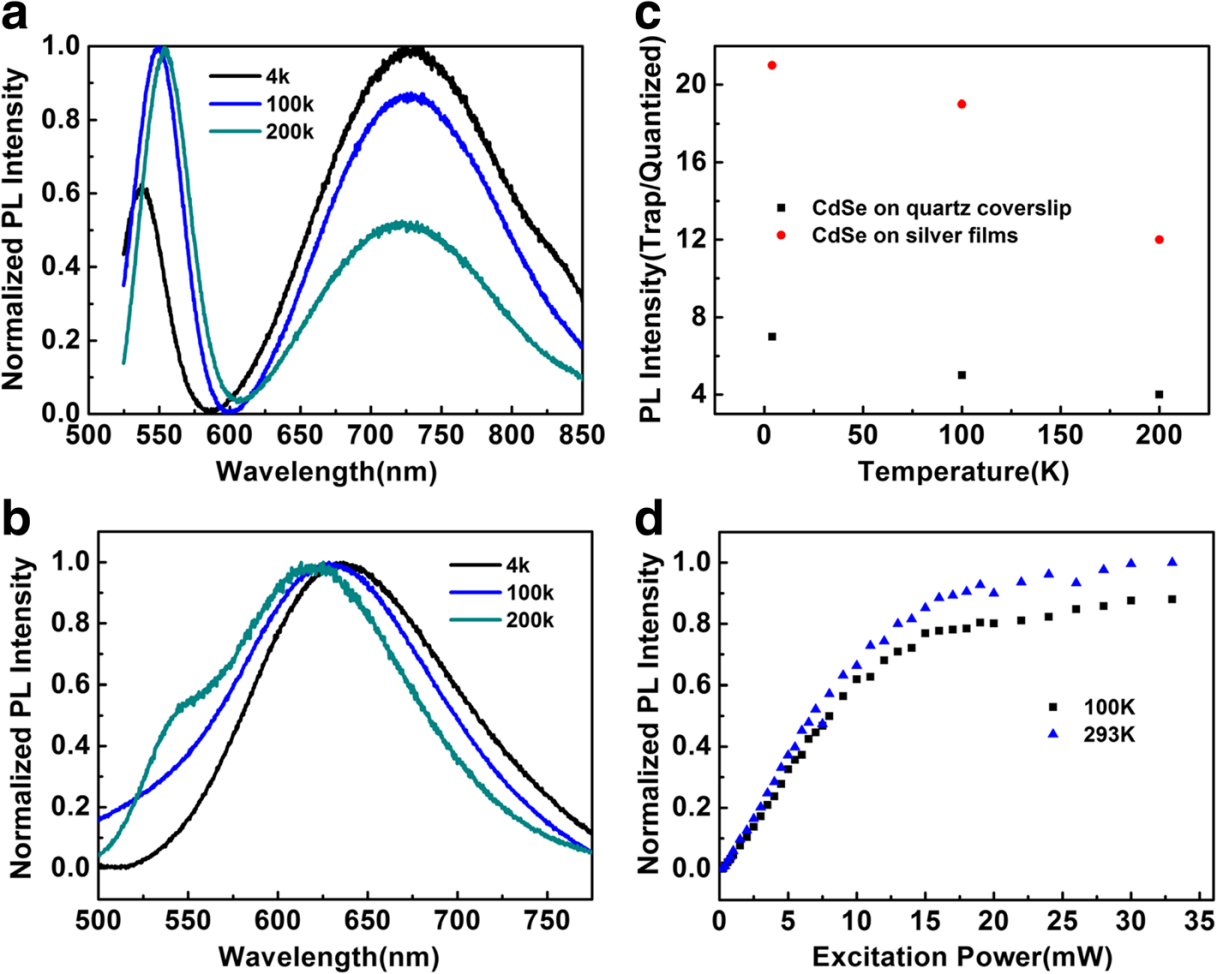
**a** Temperature-dependent PL spectra of the CdSe QDs on quartz coverslip surface and **b** on PMMA silver film. **c** The PL intensity ratio of trap states emission and quantized states emission for CdSe QDs on quartz coverslip surface and PMMA silver films. **d** Spectrally integrated PL intensities of quantized states emission on the quartz coverslip as a function of the laser power at different temperature

Temperature dependence of thermal population behavior is an important factor for the ET process from quantized states to surface states. In order to understand the PL mechanism of surface trap states, it is necessary to systematically investigate temperature effect on PL spectra and PL decay of quantized states and surface trap states, respectively. Figure 4a shows the PL spectra of quantized states and surface trap states for CdSe QDs on quartz coverslip with different temperatures (4, 100, and 200 K), respectively. From these results, the PL intensity of quantized states decreases and the blue shift is observed at low temperature (4 K), which can be explained by the temperature dependence of band gap. However, the PL intensity of trap states increases by decreasing the temperature from 200 to 4 K for CdSe QDs on quartz coverslip. Figure 4b shows the PL spectra of CdSe QDs on PMMA silver films at different temperatures (4, 100, and 200 K), respectively. Therefore, the PL measurement typically reveals that the PL intensity of surface states increases at low temperature with respect to the PL intensity of quantized states. This observation suggests that thermal population behavior causes an important impact on the fluorescence emission of surface trap states and quantized states. The red-shift indicates an evidence for the occurrence of ET process between quantized states and trap states. Moreover, ET rate is not only influenced by the dipole strengths but also influenced by wave function overlaps between the donors and acceptors. However, the two influence factors are temperature dependence. At lower temperatures, the radiative recombination rates and nonradiative recombination rates in quantized states and trap states are remarkably suppressed. Therefore, the decreased ET rate at the lower temperature qualitatively explains the decreased spectral overlap and dipole strength of the excitonic emission of QDs.

To elaborate temperature effect on PL intensity of quantized states and trap states, we compare PL intensity of quantized states with that of trap states for CdSe on the quartz coverslip and silver films, respectively. The integrated PL intensity ratio of quantized states and trap states run up to 7.1 and 23 for CdSe QDs on the quartz coverslip surface and silver films at lower temperature (4 K) as shown in Fig. 4c, respectively. From the ratio of PL intensities of quantized states and trap states, it can be concluded that the silver films further enhance the energy transfer (ET) process from the quantized states to trap states due to the surface plasmonic coupling effect. To elucidate the temperature effect on energy transfer process from quantized states to surface states, the energy transfer model consisting of core quantized state, surface state, and ground state was established [34].

In Fig. 4d, we also plot integrated PL intensities of quantized state emission for CdSe on the quartz coverslip with different excitation power. Under high-power excitation, the quantized states can absorb multiple photons and generate multiple excitons per quantized state. More importantly, the saturation power for the quantized state emission decreases with lowering the temperature to 100 K. Therefore, more excitons generated with high excitation power in quantized states easily move to surface trap states by ET process at lower temperature. Compared the PL spectra of CdSe QDs on quartz coverslip with that of CdSe QDs on the silver films, these results obviously indicate the occurrence of the ET process from the quantized states to surface trap states as a function of temperature and ET rate is obviously enhanced due to the surface plasmonic coupling effect as shown in Fig. 4b.

**Fig. 5:**
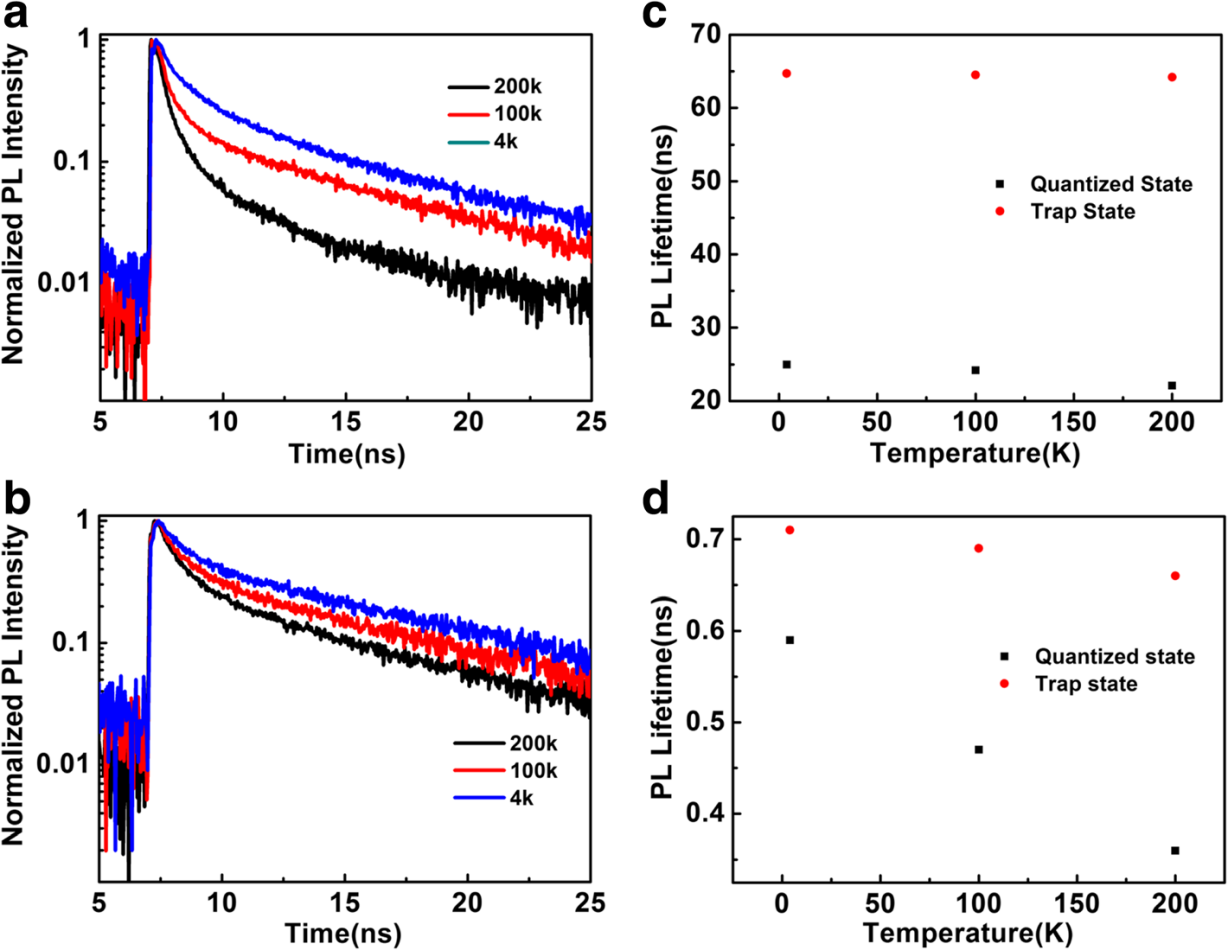
The temperature-dependent PL decay profiles of quantized states (**a**) and trap states (**b**) for CdSe QDs on the quartz coverslip at different temperature, respectively. PL lifetime of slower component (**c**) and faster component (**d**) for CdSe QDs on the quartz coverslip at different temperature, respectively

In order to investigate the temperature effect on surface states, the observed temperature-dependent PL lifetimes were monitored by the time-resolved PL measurements. Figure 5a, b shows the temperature-dependent PL decay curves of quantized states and trap states on the quartz coverslip at 4, 100, and 200 K, respectively. The PL decay curves with biexponential components consisting of radiative decay and nonradiative decay were observed. The PL lifetimes of quantized states emission can be obtained by fitting *τ*_1_ and *τ*_2_ that correspond to the slow and fast component as shown in Fig. 5a. The difference between these components is obvious at 200 K with *τ*_1_ = 22.1 ns and *τ*_2_ = 0.34 ns. As the temperature is lowered to 100 K, there is a slight change in the fast component (~0.46 ns). And the slow component with a lifetime of *τ*_1_ = 25.1 ns and the fast component with a lifetime of *τ*_2_ = 0.51 ns are observed by further cooling to 4 K. By comparing with PL lifetime of quantized states emission, the PL lifetime (~65.1 ns) of trap states can be mainly regarded as radiative decay rate due to the negligence of thermal quenching effect.

The different PL decay curves of the quantized states emission and trap states emission can be fitted as shown in Fig. 5c, d. As can be seen from the two figures, the nonradiative decay rate becomes larger at 200 K and thus, the PL decay of quantized states is faster. However, the corresponding PL decay of trap states becomes slower at lower temperature. As mentioned above, the conventional three-state model can perfectly explain the temperature effect on PL dynamic process between quantized states and surface trap states [38, 39]. Therefore, the temperature-dependent PL intensity of quantized states and surface trap states as shown in Fig. 4a, b suggests the energy transfer that occurs through thermally activated process.

## Conclusions

The temperature and wavelength dependence of energy transfer process between quantized states and trap states of CdSe QDs has been investigated by measuring PL spectra and PL decay on the quartz coverslip surface and silver films, respectively. The normalized PL intensity of the quantized states decreases at the lower temperature with respect to the trap state emission. And the enhanced ET process occurs from the quantized states to the surface trap states within the CdSe QDs, especially for CdSe QDs on the silver films at lower temperature. It can be concluded that the enhanced ET process attributes to plasmonic coupling effect at the lower temperature region and temperature dependence of thermal population behavior also plays an important role in the ET rate, especially for lower temperature.

## Abbreviations

ET: Energy transfer
NC: Nanocrystal
PL: Photoluminescence
PMMA: Polymethyl methacrylate
QDs: Quantum dots
TRPL: Time-resolved photoluminescence
